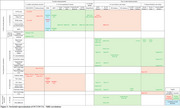# Diagnosing neurodegenerative disorders using retina as an external window: A systematic review of OCT‐MRI correlations

**DOI:** 10.1002/alz70856_098892

**Published:** 2025-12-24

**Authors:** Fei Wu, Caroline Dallaire‐Théroux, Élodie Michaud, Frédéric Bergeron, Monica Lavoie, Jean‐Paul Soucy, Ali Dirani, Robert Laforce

**Affiliations:** ^1^ Laval University Medical School, Québec, QC, Canada; ^2^ Clinique Interdisciplinaire de Mémoire, CHU de Québec‐Université Laval, Québec, QC, Canada; ^3^ Laval University, Québec, QC, Canada; ^4^ Research Chair on Primary Progressive Aphasia ‐ Fondation de la famille Lemaire, Quebec, QC, Canada; ^5^ Clinique Interdisciplinaire de mémoire, CHU de Québec ‐ Université Laval, Quebec City, QC, Canada; ^6^ Montreal Neurological Institute, McGill University, Montréal, QC, Canada; ^7^ Centre universitaire d'ophtalmologie ‐ CHU de Québec ‐ Université Laval, Québec, QC, Canada

## Abstract

**Background:**

Due to their shared embryological origin, retinal and brain tissues are affected by neurodegenerative diseases in similar ways. Optical coherence tomography (OCT) and OCT‐angiography, two non‐invasive retinal imaging modalities, have been increasingly studied as potential biomarkers for Alzheimer's disease (AD) in recent years. However, correlations between OCT/OCT‐A and neuroimaging remain understudied. We thus performed a systematic review of OCT/OCT‐A – MRI correlations in different neurodegenerative disorders associated with cognitive decline.

**Method:**

Medline, Embase, and other databases were searched from January to June 2023, using keywords related to neurodegenerative conditions and OCT/OCT‐A parameters.

**Result:**

We screened 2962 citations and 93 full‐text articles. We included 28 studies in the final review. For non‐vascular neurodegenerative diseases, layer‐specific retinal metrics, especially retinal nerve fiber layer (RNFL) thinning, and region‐specific retinal parameters (e.g. decreased foveal thickness) best correlated with changes on brain MRI. Vascular retinal biomarkers, especially reduced vessel and perfusion densities, have the unique capacity to reflect cerebrovascular lesions in vascular cognitive conditions. Both layer‐ or region‐specific retinal biomarkers and vascular retinal metrics can reflect global brain atrophy patterns. Microstructural alterations of the brain parenchyma best correlated with layer‐specific thinning of retina.

**Conclusion:**

Layer‐ or region‐specific retinal markers are better suited for non‐vascular dementias, while vascular markers more closely reflect vascular neurodegeneration. Future research must overcome several challenges including methodological heterogeneity and the complex interactions between different degenerative mechanisms. A better understanding of the associations between retinal and brain lesions could ultimately lead to the clinical use of retinal biomarkers for the early diagnosis of neurodegenerative diseases.